# The Clinical Significance of Simultaneous IL-17A and IL-17F Blockade in Psoriasis Non-Responding to Anti-IL17A Therapy

**DOI:** 10.3390/jcm12010035

**Published:** 2022-12-21

**Authors:** Georgios Kokolakis, Kamran Ghoreschi

**Affiliations:** 1 Psoriasis Research and Treatment Centre, Charité—Universitätsmedizin Berlin, Corporate Member of Freie Universität Berlin and Humboldt-Universität zu Berlin, 10117 Berlin, Germany; 2 Department of Dermatology, Venereology and Allergology, Charité—Universitätsmedizin Berlin, Corporate Member of Freie Universität Berlin and Humboldt-Universität zu Berlin, 10117 Berlin, Germany

**Keywords:** psoriasis, scalp psoriasis, psoriasis arthritis, bimekizumab, IL-17, IL-23, TNFα

## Abstract

The better understanding of the immunopathogenesis of psoriasis has led to the development of highly efficacious targeted therapies with favorable safety profiles. Among them, the class of Interleukin (IL)-17 antibodies are well established for the treatment of psoriasis, psoriatic arthritis and axial spondyloarthritis. Bimekizumab is a new antibody that simultaneously neutralizes IL-17A and IL-17F. We present two patients with psoriasis, who lost response to several biologics, among them IL-17 antagonists such as secukinumab, ixekizumab or brodalumab. Besides plaque-type psoriasis, patients also had psoriasis in hard-to-treat areas such as scalp and groins or psoriatic arthritis. Remarkably, both patients already responded to the therapy with bimekizumab 4 weeks after the first injection and, one year thereafter, both patients sustained PASI100. No side effects were observed. The fast response to bimekizumab emphasizes the crucial role of IL-17F in the pathogenesis of psoriasis. Besides, due to the new mechanism of action, non-responders to other anti-IL-17 therapies could benefit when switched to bimekizumab.

## 1. Introduction

Psoriasis is a common inflammatory skin disease affecting 3% of the European population. The underlying immunopathogenic mechanisms have been well described in the last decade. Therefore, new targeted therapies have been developed. Psoriasis is considered as a T-cell mediated inflammatory disease, where T-helper (Th)-17 cells play the central role. Almost twenty years ago, monoclonal antibodies or fusion proteins selectively blocking Tumor Necrosis Factor alpha (TNFα) have strikingly improved the therapeutic response of psoriasis. TNF blockers show an excellent response and tolerability compared to previous conventional non-selective immunosuppressants such as cyclosporine A or methotrexate [1]. Even better results and more beneficial safety profiles could be observed when directly blocking the psoriasis-driving Th-17 response by neutralizing either IL-23 or IL-17 [2].

While IL-23 is produced by antigen-presenting cells such as macrophages and dendritic cells, IL-17 is produced by T cells, innate lymphoid cells and mast cells. Interleukin-17 is a cytokine family consisting of IL-17A, IL-17B, IL-17C, IL-17D, IL-17E and IL-17F. Besides IL-17AA-FF, -CC, -EE homodimers, IL-17AF heterodimers are also formed (2). IL-17AA, IL-17FF and to a lesser extent IL-17AF are the dominant IL-17 cytokines in psoriasis plaques, that transmit inflammatory signals between skin resident or skin infiltrating T-cells and either keratinocytes or neutrophils. IL-17 activates keratinocytes, which on their turn also produce inflammatory cytokines, in this way stimulating skin resident T-cells and amplifying a vicious circle of inflammatory interaction between the skin and immune system. The neutralization of IL-17A interrupts this interaction and has been proven as a fast and efficacious therapy for psoriasis [2]. Secukinumab and Ixekizumab were the first IL-17A monoclonal antibodies that were developed and established with great success in psoriasis treatment [3,4]. Brodalumab is a monoclonal antibody against the IL-17RA subunit of the IL-17 receptor approved for plaque-type psoriasis, which inhibits the signals derived from homo- or heterodimers of IL-17A, IL-17F, IL-17C or IL-17E [5,6]. The newest development of the class of IL-17 inhibitors is bimekizumab, a monoclonal antibody simultaneously targeting IL-17AA, IL-17AF and IL-17FF [7]. The even superior efficacy of bimekizumab compared to secukinumab emphasizes the underestimated role of IL-17F in the evolution of psoriatic plaques [8].

In the present study, we report the first two psoriasis patients treated with bimekizumab after its European Medicines Agency approval who failed to treatment with anti-IL-17A therapy but responded to the blockade of IL-17A/IL-17F.

## 2. Case Reports

**Figure 1:**
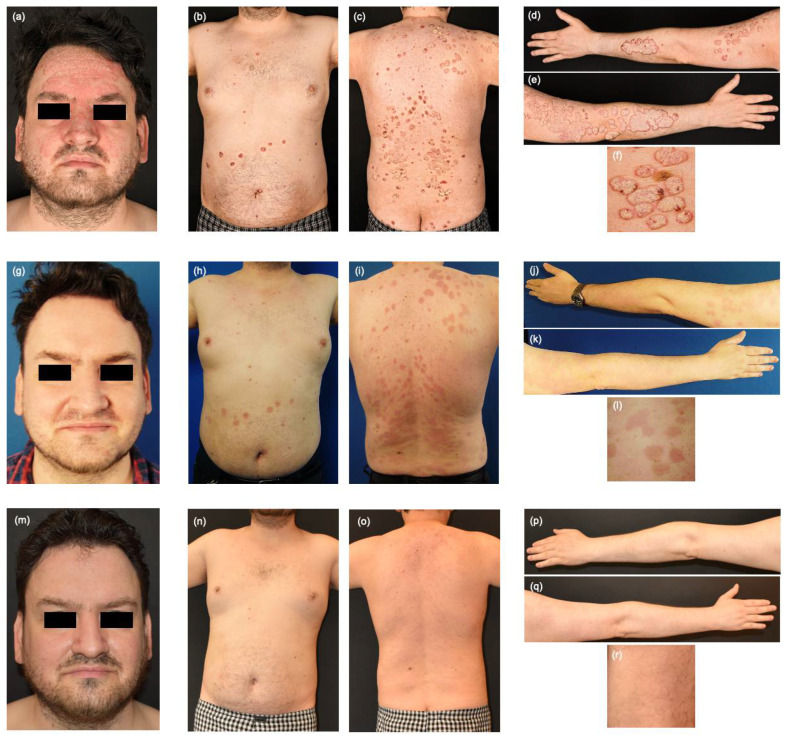
Widespread well-demarcated erythematosquamous plaques on the face and hairy scalp, trunk and upper extremities (**a**–**e**) and representative plaques of the lower extremities (**f**) after 3 months of therapy with ixekizumab, topical therapy and UVB phototherapy (PASI 16.4; DLQI 16). Complete remission of psoriasis on the face (**g**) and residual erythematous maculae and descent infiltrated plaques with fine scaling on the trunk, upper and lower extremities (**h**–**l**) 4 weeks after administration of bimekizumab 320mg (PASI 7.8; DLQI 6). Maintenance of complete remission of face psoriasis (**m**) and clearance of psoriasis of the body (**n**–**r**) after 12 months of treatment (PASI 0; DLQI 0).

A 38-year-old male patient presented with severe psoriasis for 21 years affecting all body regions including scalp and groins (PASI 28.8; DLQI 16). Besides psoriasis, depression and hypothyreodism were marked in his medical history. Both parents also suffered from psoriasis. Ixekizumab had been initiated 10 weeks before his visit in our clinic. The patient was admitted in our in-patient clinic, where he received intensive local therapy with dithranol ointment, talcitriol and topical steroids as well as 13 sessions of UVB narrow-band (311 nm) phototherapy. Ixekizumab was continued until week 12. The skin condition moderately improved (PASI 16.4; DLQI 16) after 3 months of ixekizumab, and the scalp was still prominently affected (Figure 1a–f). Previous systemic therapies included methotrexate, secukinumab, adalimumab, ustekinumab, brodalumab, tildrakizumab, risankizumab and etanercept. So far, the best results were achieved with secukinumab or adalimumab, where patient lost response after approximately 3 years of each treatment. All other biologics were ceased shortly after the induction because of primary loss of response or non-response. Due to the patient’s long experience with multiple biologics and the good response of secukinumab, we decided to stay in the class of IL-17 inhibitors and introduce bimekizumab 320 mg s.c. as a new biologic treatment three weeks after the last administration of ixekizumab. Bimekizumab 320 mg was injected s.c. every 4 weeks for the first 16 weeks and then every 8 weeks. Already 4 weeks after the first injection of the IL-17A/IL-17F antagonist, the patient’s skin condition improved dramatically. Psoriasis plaques of the forehead and scalp almost cleared and PASI and DLQI were reduced to 7.8 and 6, respectively (Figure 1g–l). No side effects were observed. In the one-year follow-up visit the patient appeared with clear skin (PASI 0) and no impact of psoriasis in his quality of life (DLQI 0) (Figure 1m–r). No candida infections or other side effects were observed.

**Figure 2:**
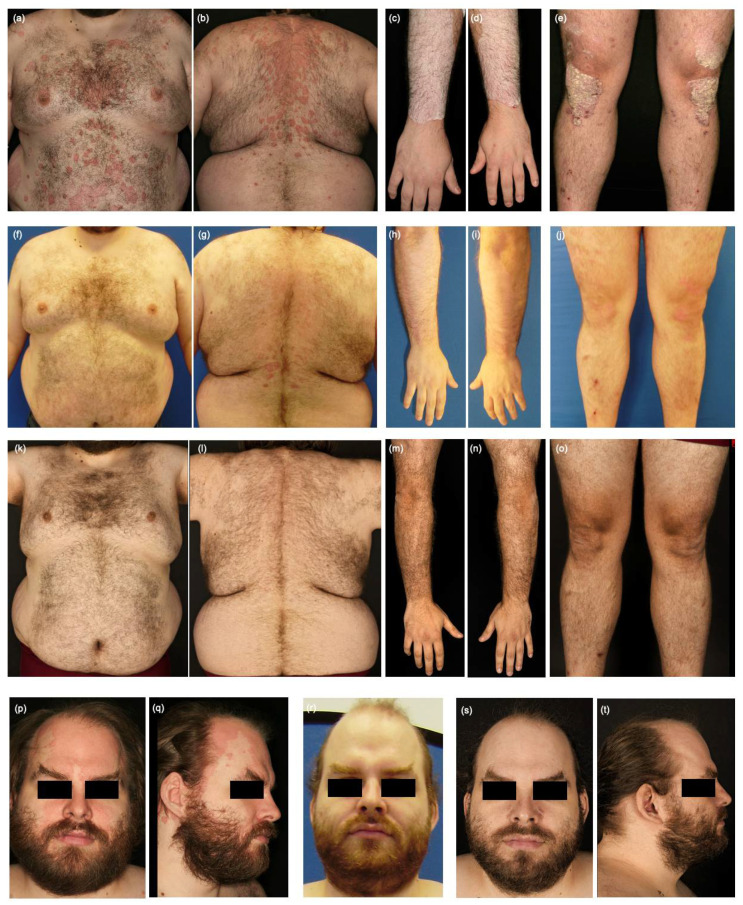
Psoriasis plaques on the trunk (**a**,**b**) and the upper and lower extremities, predominantly on dorsal forearms (**c**–**e**) marked by confluent red, well-demarcated, scaly plaques under risankizumab for two years (PASI 19.5; DLQI 21). Improvement of psoriasis of the trunk and the extremities 4 weeks (**f**–**j**) (PASI 3.7; DLQI 7) and 12 months (**k**–**o**) (PASI 0; DLQI 0) after the first injection of bimekizumab 320 mg. Resolution of scalp psoriasis after 4 weeks (**r**) and 12 months (**s**,**t**) of therapy compared to baseline (**p**,**q**).

Our second case is about a 35-year-old male obese patient (BMI 44.3) with severe psoriasis since the age of ten. When visiting our department, he presented as a secondary non-responder to risankizumab after a total of 2 years of therapy (PASI 19.5; DLQI 21) (Figure 2a–e,p,q). The patient also suffered from psoriatic arthritis, enthesitis and inflammatory back pain (HLA-B27 negative), which worsened in the last two years. However, the diagnosis of axial spondyloarthritis was not confirmed. He reported morning stiffness of more than 1 h and pain in carpal and metacarpal joints, fingers, lower back and Achilles’s tendons bilaterally. Depression and asthma were among his medical history. Previously, the patient was treated with topicals, phototherapy, non-steroidal anti-inflammatory drugs, methotrexate, etanercept, adalimumab, ustekinumab, secukinumab and guselkumab. Biological therapies had to be switched because of secondary loss of response. Since two IL-23 antibodies, guselkumab and risankizumab, sequentially failed in long-term disease control, we decided to switch the class of biologics and started bimekizumab 320 mg. Bimekizumab was injected according to the label with 8 week intervals after the monthly induction in the initial 16 weeks. Four weeks after the first administration of bimekizumab, his skin condition rapidly improved (PASI 3.7; DLQI 7) (Figure 2f–j,r). Likewise, patient’s joint complaints significantly improved. No morning stiffness or swelling of the joints were reported, only a mild pain in the Achilles tendons persisted. In the annual control examination, the patient presented with PASI 0 and DLQI 0 (Figure 2k–o,s,t) and no joint or back pain. No treatment-specific adverse events, especially candida infections, were reported. The mobility of the patient improved so that he achieved a BMI 40.9 through physical activity and proper nutrition. 

## 3. Discussion

The development of new classes of biologics apart from TNF inhibitors expanded the therapeutic possibilities for psoriasis. The selective blockade of IL-17A through secukinumab or ixekizumab, causing a simultaneous neutralization of IL-17AA homodimers and IL-17AF heterodimers, has shown a fast and constant efficacy in the treatment of plaque-type psoriasis, nail psoriasis and psoriatic arthritis [3,4,9,10]. Likewise, brodalumab, a monoclonal antibody against the IL-17RA subunit of the IL-17 receptor has been approved for psoriasis and has shown convincing results in psoriatic arthritis [6,11,12]. In the case of our patients, blocking IL-17A or the receptor unit IL-17-RA failed to control psoriasis. In the first case, the patient was a primary non-responder of ixekizumab and brodalumab, and secondary non-responder of secukinumab. Even if the actions of IL-17F were neutralized by blocking the IL-17RA receptor with brodalumab, only limited efficacy on his psoriasis was observed. The patient rapidly responded to the first injection of bimekizumab. It can be assumed that the high binding affinity of bimekizumab to IL-17F provides a stronger inhibition of IL-17AF, and -FF signaling than the receptor antagonist brodalumab [13], which led to this remarkable clinical outcome. The prominent role of IL-17F in the evolution of the psoriatic plaques has been well investigated. IL-17F expression in psoriatic plaques seems to be significantly higher than IL-17A. Besides, much higher levels of IL-17F than IL-17A protein have been detected in the serum of patients with psoriasis [14].

Similarly, the second patient lost response of secukinumab after 12 months of treatment. Following that, the patient was sequentially treated with two IL-23 antibodies, guselkumab and risankizumab, with sufficient response for up to two years. Guselkumab and risankizumab strongly impair the differentiation of pathogenic Th17 cells, the main producers of the effector cytokine IL-17A and IL-17F [2]. However, this patient also showed a rapid and excellent response to bimekizumab, demonstrating that simultaneous IL-17A/IL-17F blockade is a stronger inhibitor of the Th17 pathway. Our report indicates that bimekizumab can be effective in anti-IL-23 or anti-IL-17A non-responders. Recently, two years data on high efficacy of bimekizumab together with a favorable safety profile was reported in patients switching from ustekinumab, an IL-12/IL-23 antibody [15]. Besides, plaque-type psoriasis, psoriatic arthritis and enthesitis also improved under bimekizumab. Although bimekizumab is not yet approved for the treatment of psoriatic arthritis, results of recent clinical phase 2 trials have demonstrated clinical efficacy of bimekizumab also in this indication [16].

Both patients were experienced on more than two biological therapies. Previous biologic use has been reported to affect treatment response and drug survival of subsequent biologics [17]. In a phase 3 clinical trial, bimekizumab was highly efficacious in psoriasis in a cohort with 44% biologic-experienced patients, among them also anti-IL-17 non-responders [7]. In case of incomplete response, the dosing of bimekizumab for the treatment of plaque-type psoriasis provides the flexibility to shorten the injection intervals from 8 weekly to 4 weekly in patients with a body weight of more than 120 kg (EMA) [18].

## 4. Conclusions

In conclusion, the dual neutralization of IL-17A and IL-17F can be efficacious for fast and sustainable management of psoriasis in a real world setting, even in special skin localizations such as the scalp or the groins, when the blocking of IL-17A, IL17-RA or IL-23 has previously failed.

## Data Availability

The data presented in this study are available on request from the corresponding author. The data are not publicly available due to patients’ privacy.
